# Understanding the dual-task costs of walking: a StartReact study

**DOI:** 10.1007/s00221-020-05817-8

**Published:** 2020-04-30

**Authors:** Jorik Nonnekes, Valeria Dibilio, Claudia Barthel, Teodoro Solis-Escalante, Bastiaan R. Bloem, Vivian Weerdesteyn

**Affiliations:** 1 Department of Rehabilitation, Radboud University Medical Centre, Donders Institute for Brain, Cognition and Behaviour, Nijmegen, The Netherlands; 2 Sint Maartenskliniek Research, Nijmegen, The Netherlands; 3 IRCCS Centro Neurolesi Bonino Pulejo, Messina, Italy; 4 Department of Neurology, Radboud University Medical Centre, Donders Institute for Brain, Cognition and Behaviour, Centre of Expertise for Parkinson and Movement Disorders, Nijmegen, The Netherlands; 5 SRH University of Applied Health Sciences, Gera, Campus Stuttgart, Stuttgart, Germany

**Keywords:** Startle, StartReact, Gait, Dual-task

## Abstract

The need to perform multiple tasks more or less simultaneously is a common occurrence during walking in daily life. Performing tasks simultaneously typically impacts task performance negatively. Hypothetically, such dual-task costs may be explained by a lowered state of preparation due to competition for attentional resources, or alternatively, by a ‘bottleneck’ in response initiation. Here, we investigated both hypotheses by comparing ‘StartReact’ effects during a manual squeezing task under single-task (when seated) and dual-task (when walking) conditions. StartReact is the acceleration of reaction times by a startling stimulation (a startling acoustic stimulus was applied in 25% of trials), attributed to the startling stimulus directly releasing a pre-prepared movement. If dual-task costs are due to a lowered state of preparation, we expected trials both with and without an accompanying startling stimulus to be delayed compared to the single-task condition, whereas we expected only trials without a startling stimulus to be delayed if a bottleneck in response initiation would underlie dual-task costs. Reaction times of the manual squeezing task in the flexor digitorum superficialis and extensor carpi radialis muscle were significantly delayed (approx. 20 ms) when walking compared to the seated position. A startling acoustic stimulus significantly decreased reaction times of the squeezing task (approx. 60 ms) both when walking and sitting. Dual-task costs during walking are, therefore, likely the result of lowered task preparation because of competition for attentional resources.

## Introduction

Dual tasking is the simultaneous performance of two attention-demanding tasks with different goals, whereby one task can be denoted as the primary and the other as the secondary task (Beauchet and Berrut 2006). When walking under daily life situations, we often perform such secondary tasks, for example when talking to someone or when handling a mobile phone. Under these circumstances, gait can be seen as the primary task, and talking or handling a mobile phone as the secondary task. Performing two tasks simultaneously during gait impacts task performance in a negative manner (Sparrow et al. 2002; Woollacott and Shumway-Cook 2002). Two competing hypotheses have been suggested for the mechanism underlying these dual-task effects. First, the negative influence of dual-task performance may be due to a lowered state of preparation of either task due to competition for attentional resources (Maslovat et al. 2015). Alternatively, dual-task costs may be explained by a response initiation ‘bottleneck’ (Bratzke et al. 2009), whereby it is difficult to produce multiple responses simultaneously. Evidence for the bottleneck hypothesis came from studies that reported additional dual-task constraints when the primary and secondary task had the same effector (e.g., when both were manual in nature) compared to tasks where the primary and secondary task had different effectors (e.g., vocal and manual in nature) (Lee and Elliott 1986). Based on these assumptions, one would expect that dual-task constraints during walking are due to competition for attentional resources, as the primary and secondary tasks typically have different effectors (e.g., talking while walking, or handling a mobile phone). In this study, we apply the StartReact paradigm to study the mechanism underlying dual-task costs during walking. Reaction times can be accelerated by a startling acoustic stimulus, a phenomenon described in the seminal work by Valls-Sole, Rothwell, and colleagues (Valls-Sole et al. 1999), and which is now known as the StartReact effect. Acceleration of reaction times by a startling stimulus is a brainstem-medicated process that is limited to tasks that allow for motor preparation, i.e., acceleration of reaction times is commonly absent during choice reaction tasks (Carlsen et al. 2004). The StartReact effect is attributed to the startling stimulus directly releasing a pre-prepared movement (Valls-Sole et al. 1999, 2008; Rothwell 2006; Nonnekes et al. 2014b). Here, we evaluated StartReact effects when a manual squeezing task was applied under single-task conditions (i.e., when seated) or dual-task conditions (i.e., when walking). We hypothesized that if dual-task costs are due to a lowered state of preparation, both trials with and without an accompanying startling stimulus would be delayed compared to the single-task condition, whereas we expected only trials without a startling stimulus to be delayed if a bottleneck in response initiation would underlie dual-task costs.

## Materials and methods

### Participants

Ten healthy adults (7 women, mean 27 years, range 24–31 years) participated. None of the participants suffered from hearing, neurological, or motor disorders that could interfere with their performance during the experiments. None of the subjects used medication that could interfere with reaction times. All subjects gave their written informed consent prior to participation, and the experiment was performed in accordance with the Declaration of Helsinki and approved by the local ethics committee (medical-ethical committee Arnhem-Nijmegen).

### Experimental setup and protocol

Participants were instructed to hold a rubber ball (6 cm in diameter) in their dominant hand (all participants were right-handed). They had to perform a simple reaction task that involved squeezing the ball as fast as possible in response to an auditory tone (70 dB, sound pressure level). Participants performed the reaction task under two conditions: (1) while sitting relaxed on a chair without arm rests; and (2) while continuously walking at a self-selected comfortable speed along a 20-m circuit. The order of the conditions was counterbalanced across subjects. In each condition, participants performed 16 trials. In 25% of these trials the auditory tone was randomly replaced by a startling acoustic stimulus (SAS, 115 dB sound pressure level). Both the auditory tone and SAS were given through binaural earphones (Sennheiser DS218) and consisted of 50 ms of white noise. The time between the trials varied between 15 and 45 s, auditory stimuli were not given when turning. There was no warning signal before the auditory tone. Matlab software was used to control the trials. During the dual-task condition, we instructed participants that it was of importance that they continued steady-state walking, despite the presence of the reaction time task. Hence, walking was regarded as the primary task.

### Data collection

Muscle activity was measured using surface electromyography (EMG) of the dominant flexor digitorum superficialis (FDS) muscle, extensor carpi radialis (ECR) muscle, and left sternocleidomastoid (SCM) muscle (ZeroWire by Aurion, Italy; 2000 Hz). Self-adhesive Ag–AgCl electrodes (Tyco Arbo ECG) were placed approximately 2 cm apart and longitudinally on the belly of each muscle, according to Seniam guidelines (Hermens 1999). To assess response onset, an analog pressure sensor (ZeroWire by Aurion, Italy) was placed inside the ball. All signals were recorded at 2000 Hz. For further processing, EMG signals were full-wave rectified and low-pass filtered at 30 Hz (zero-lag, second-order Butterworth filter), which was done after data collection.

### Data analysis

Two reaction time parameters were assessed: EMG reaction time and sensor reaction time. Muscle onset latencies were determined using a semi-automatic computer algorithm that selected the first instant at which the EMG activity exceeded a threshold of 2 SD above the background activity, as calculated over a 500 ms period just prior to the auditory ‘go’ signal (Nonnekes et al. 2013, 2014a, b). Onsets were first selected by the computer algorithm, and then visually approved and (when necessary) corrected. Onset latencies were calculated for each trial separately. Response onset as recorded by the pressure sensor was determined in the same manner. For each trial in which an SAS was applied, we determined whether a startle reflex occurred. A startle reflex was defined as a short latency response in the SCM muscle, starting within 130 ms following the SAS (Nonnekes et al. 2013, 2014a, b). Six trials (out of 320 trials, 2 SAS and 4 non-SAS) were not included in the analysis because of errors made by the participants.

### Statistical analysis

The effects of SAS (SAS–non-SAS) and Condition (standing–walking) on the individual reaction times were evaluated using general linear mixed-effects (GLME) models. The models included independent linear terms for fixed effects of SAS, Condition, and the interaction SAS × Condition. These linear terms were controlled for random effects of participants. In addition, the GLME models included a random intercept (offset) per participant. The factors SAS and Condition were treated as categorical variables. There were three GLME models using all available trials for the reaction times obtained from the FDS and ECR muscles and the pressure sensor; and three additional GLME models using only those trials with a concurrent response in the SCM muscle. These additional models were necessary, because SAS trials with SCM responses may have different reaction times than those trials without SCM response (Leow et al. 2018). Differences in the occurrences of startle responses in the SCM muscle between sitting and standing were analyzed using Pearson’s Chi-square tests. The alpha level was set at 0.05.

## Results

**Fig. 1:**
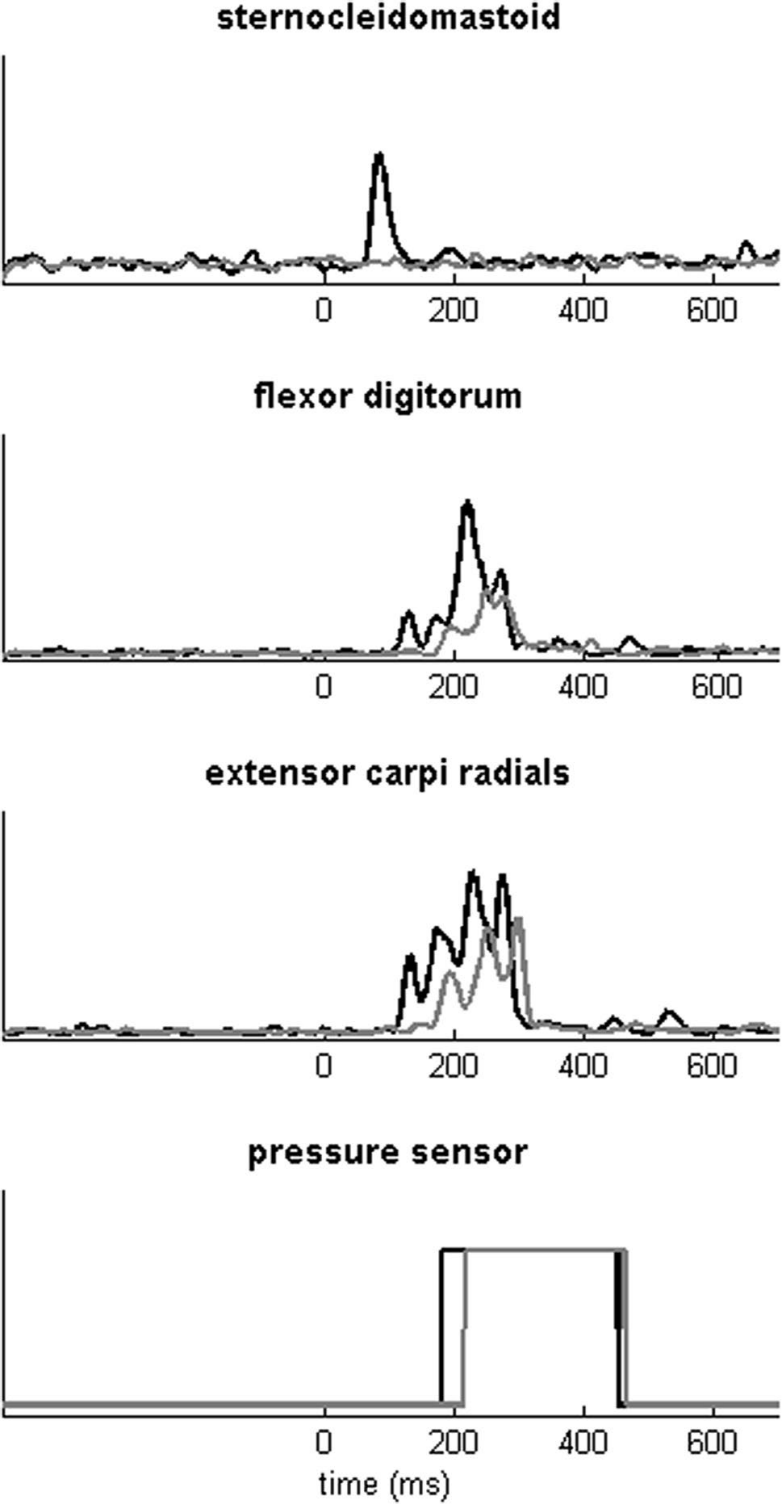
EMG traces of a representative participant during a trial with and without an SAS. Black lines are trials with an SAS; gray lines are trials without a SAS

**Fig. 2:**
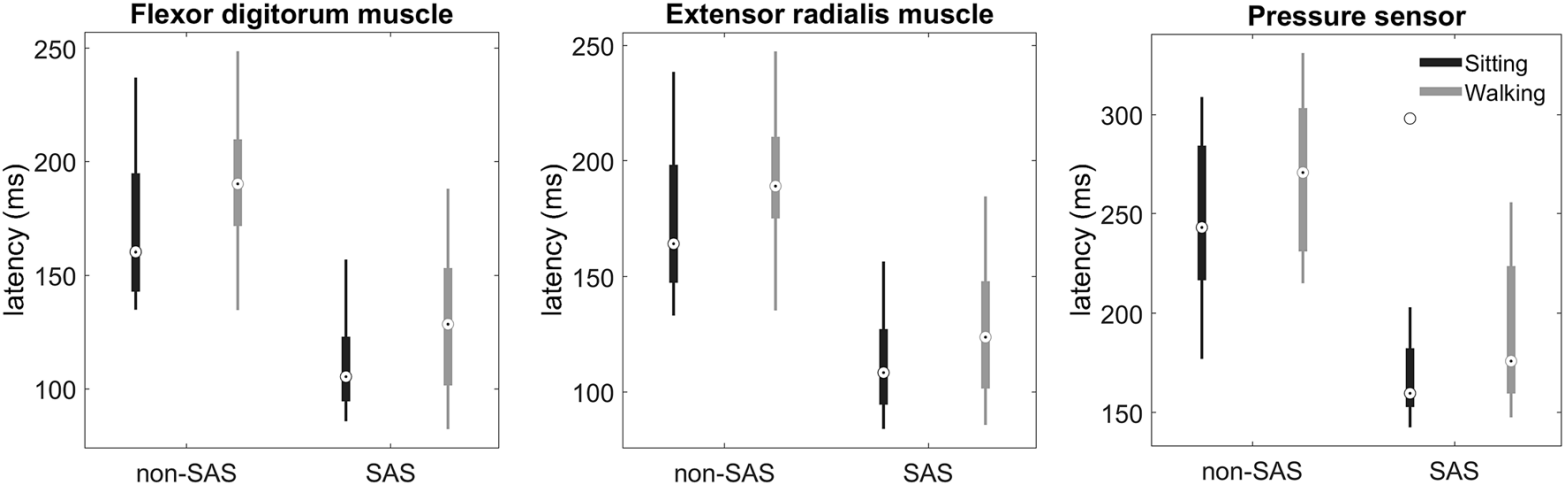
Distribution of participant’s mean reaction times. The boxplots show the reaction time estimated from flexor and extensor muscles, as well as the pressure sensor. General linear mixed-effects models showed significant effects of stimulus (non-SAS vs. SAS) and condition (sitting vs. walking), but no interaction between stimulus and condition. The open circles with a black dot mark the group median response latency, and the top and bottom edges of the box indicate the 25th and 75th percentiles of the mean reaction times; the whiskers extend to the maximum and minimum value (excluding outliers). Outliers are plotted as open circles

When squeezing the ball, the FDS and ECR muscles were simultaneously activated (Fig. 1). Irrespective of whether the participants were sitting or walking, the SAS reduced FDS onset latencies (SAS: coef. − 57.6, SE: 8.9, CI: [− 75.1, − 40.1], *t*(311) =  − 6.5, *p* < 0.001; SAS × condition: *t*(311) =  − 0.4, *p* = 0.689; Fig. 2). FDS onset latencies were on average 22 ms longer when walking compared to sitting (condition: coef. 22.6, SE: 8.3, CI: [6.2, 39.0], *t*(311) = 2.7, *p* = 0.007).

This pattern was also found for ECR onset latencies (Fig. 2). A SAS significantly reduced the onset latencies during sitting and walking, without significant differential effects across these conditions (SAS: coef. − 58.4, SE: 8.8, CI: [− 75.8, − 41.0], *t*(311) =  − 6.6, *p* < 0.001; SAS × condition: *t*(311) =  − 0.5, *p* = 0.601). Onset latencies were on average 21 ms longer when walking compared to performing the reaction task in a seated position (condition: coef. 21.4, SE: 7.9, CI: [5.9, 36.9], *t*(311) = 2.7, *p* = 0.007).

The same pattern was also observed for the response latency determined using the pressure sensor. A SAS reduced the onset latencies in the seated and the walking conditions (SAS: coef. − 66.2, SE: 11.7, CI: [− 89.3, − 43.2], *t*(306) = 11.7, *p* < 0.001; SAS × condition: *t*(306) = − 0.9, *p* = 0.358). Reaction times were 26 ms longer while walking compared to the seated condition (condition: coef. 26.0, SE: 9.0, CI: [8.2, 43.8], *t*(306) = 2.9, *p* = 0.004).

The occurrence of startle reflexes in the SCM muscle during trials with a SAS was significantly higher in the seated condition (78% of trials with a SAS; 9 out of 10 participants showed at least one startle reflex in the SCM muscle) compared to the walking condition (53% of trials with a SAS, *p* = 0.038; *χ*^2^(1) = 4.28, *p* = 0.038; 7 out of 10 participants displayed a SCM startle reflex in at least one trial). The alternative GLME models (including 287 out of 314 trials; 51 SAS and 236 non-SAS) using only the SAS trials with SCM responses showed the same effects as those reported above. Thus onset latencies were shorter when the SAS was applied [FDS: coef. − 63.7, *t*(284) = − 6.5, *p* < 0.001; ECR: coef. − 65.0, *t*(284) = − 6.7, *p* < 0.001; Sensor: coef. − 78.7, *t*(280) = − 6.5, *p* < 0.001]; and reaction times were longer in the walking than in the sitting condition [FDS: coef. 22.7, *t*(284) = 2.5, *p* = 0.011; ECR: coef. 21.5, *t*(284) = 2.5, *p* = 0.011; Sensor: coef. 26.0, *t*(280) = 2.9, *p* = 0.003]; but no significant SAS × Condition interaction effect was present (FDS: *t*(284) = − 0.37, *p* = 0.714; ECR: *t*(284) = − 0.5, *p* = 0.589; Sensor: *t*(280) = − 0.2, *p* = 0.819).

## Discussion

We investigated the mechanism underlying dual-task costs during gait, by comparing StartReact effects on a manual squeezing task performed under single-task conditions (when seated) and dual-task conditions (when walking). A startling acoustic stimulus (SAS) strongly reduced onset latencies of the manual squeezing task, both when seated and when walking. However, onset latencies were slightly—but significantly—delayed when walking compared to the seated position, both for trials with and without a startling acoustic stimulus. The findings were found when analyzing all SAS trials, and also when only including SAS trials with a startle reflex in the SCM muscle.

Our findings suggest that dual-task costs during walking are the result of a lowered state of task preparation because of competition for attentional resources. Our results are line in with those of earlier work (Maslovat et al. 2015) that investigated the mechanisms underlying dual-task costs using a paradigm where the primary task was either motor (pursuit tracking) or cognitive (counting backward) in nature, and where the secondary task involved an upper extremity motor reaction time task. Although reaction times were reduced by an SAS, both trials with and without a startling stimulus were moderately delayed in single-task conditions compared to dual-task conditions. In their study, single-task performance was always assessed following dual-task performance, and reduced reaction times during single-task conditions may, therefore, have resulted from practice effects. As we counterbalanced single- and dual-task conditions, our results show that it is unlikely that practice effects underlie differences in reaction times between single- and dual-task conditions. The present study adds to the literature in two ways. First, we show that the findings of Maslovat and colleagues also apply to dual-task gait conditions. Second, as steady-state gait is regarded as a largely automatic task (and as less attention demanding than pursuit tracking or counting backwards), our study shows that their findings are also applicable to more automatic motor tasks.

Our findings may be explained by the neural activation framework (Hanes and Schall 1996), in which response initiation occurs once a group of neurons responsible for the movement reaches a threshold of neural activation (Wickens et al. 1994). In this model, task preparation involves an increase in the activation levels of neurons to a sub-threshold level, such that less time is required to raise activations levels to the initiation thresholds. Our data suggest that during dual-task gait conditions, there are reduced attentional resources for the secondary task (in our study a squeezing task, which likely involves cortico-striatal loops), resulting in a lowered state of task preparation (less increase in neural activation levels of the involved cortico-striatal loops) and subsequently increased reaction times (Woollacott and Shumway-Cook 2002).

Interestingly, in line with Maslovat and colleagues, we also found that startle reflexes in the SCM muscle were more frequent in the single-task condition compared to the dual-task condition, which may reflect ‘reduced state of readiness’ during dual-task conditions (Maslovat et al. 2015). In line with this hypothesis, it has been found that the probability of eliciting a startle response is higher when participants are fully prepared for a voluntary response compared to less prepared conditions (Carlsen and MacKinnon 2010). The mechanism underlying more frequent startle reflexes during single-task conditions compared to dual-task conditions, however, remains to be unraveled, as startle reflexes arise from the pontomedullary reticular formation (Valls-Sole et al. 2008), whereas the squeezing task likely involves cortico-striatal-loops. An increase of neural activation levels in cortico-striatal loops may have downstream effects on the pontomedullary reticular formation (increasing neural activation levels), explaining the more frequent startle reflexes in the single-task condition compared to the dual-task condition. However, this is speculative, and this issue should be topic for future studies.

Our findings suggest that even unconstrained walking in healthy young subjects requires substantial attentional resources. The presented framework would predict that in people with reduced attentional resources or in people increased attentional demands for performing the requested secondary task (e.g., elderly people with neurological conditions such as stroke or Parkinson’s disease), there is more competition for attention resources, resulting in a lowered state of task preparation and higher dual-task costs. Indeed, higher dual-task costs have been found in elderly persons, in persons after stroke and in people with Parkinson’s disease (Sparrow et al. 2002; Chen et al. 2013; Raffegeau et al. 2019). For example, healthy elderly people have the same baseline reaction times compared to healthy young participants, but a larger increase in reaction times compared to healthy young subjects when performing the same reaction time task during walking (Sparrow et al. 2002). Another example is a larger deterioration of a secondary cognitive task in stroke patients compared to healthy age-matched controls, when simultaneously performing a primary obstacle avoidance task on a treadmill (Smulders et al. 2012). Interestingly, success rates of obstacle avoidance did not differ between single- and dual-task condition in both healthy controls and stroke patients, whereas the other studies also reported a deterioration of the primary gait task (Haggard et al. 2000; Hyndman et al. 2006).

The fact that we did not evaluate whether gait changed (e.g., by looking at the spatiotemporal parameters such as gait speed or gait variability) when performing a dual-task condition compared to walking without performing a secondary task is a limitation of the present study. In addition, it would have been stronger if we had measured sternocleidomastoid activity bilaterally, and included more trials (although we found significant effects with these limited number of trials). Moreover, this study would have been stronger if we had evaluated dual-task costs not only during unconstrained walking, but also when walking over uneven terrain or during obstacle avoidance. We hypothesize that reaction times—both in trials with and without a startling stimulus—may further increase during these attention-demanding gait conditions, but this may be a topic for future studies.
